# Cyanidin-3-*O*-Glucoside-Rich Haskap Berry Administration Suppresses Carcinogen-Induced Lung Tumorigenesis in A/JCr Mice

**DOI:** 10.3390/molecules25173823

**Published:** 2020-08-22

**Authors:** Madumani Amararathna, David W. Hoskin, H. P. Vasantha Rupasinghe

**Affiliations:** 1 Department of Plant, Food, and Environmental Sciences, Faculty of Agriculture, Dalhousie University, 50 Pictou Road, Truro, NS B2N 5E3, Canada; madu.ama@dal.ca; 2 Department of Pathology, Dalhousie University, Halifax, NS B3H 4R2, Canada; d.w.hoskin@dal.ca; 3 Department of Microbiology and Immunology, Dalhousie University, Halifax, NS B3H 4R2, Canada

**Keywords:** anthocyanin, tobacco-specific nitrosamine, carcinogenesis, cell proliferation, cancer chemoprevention, lung cancer

## Abstract

In our previous study, we demonstrated that cyanidin-3-*O*-glucoside (C3G)-rich haskap (*Lonicera caerulea* L.) berry extracts can attenuate the carcinogen-induced DNA damage in normal lung epithelial cells *in vitro*. Here, the efficacy of lyophilized powder of whole haskap berry (C3G-HB) in lowering tobacco-specific nitrosamine, 4-(methylnitrosamino)-1-(3-pyridyl)-1-butanone, (NNK)-induced lung tumorigenesis in A/JCr mice was investigated. Three weeks after daily oral administration of C3G-HB (6 mg of C3G in 0.2 g of C3G-HB/mouse/day), lung tumors were initiated by a single intraperitoneal injection of NNK. Dietary C3G-HB supplementation was continued, and 22 weeks later, mice were euthanized. Lung tumors were visualized through positron emission tomography (PET) and magnetic resonance imaging (MRI) 19 weeks after NNK injection. Dietary supplementation of C3G-HB significantly reduced the NNK-induced lung tumor multiplicity and tumor area but did not affect tumor incidence. Immunohistochemical analysis showed reduced expression of proliferative cell nuclear antigen (PCNA) and Ki-67 in lung tissues. Therefore, C3G-HB has the potential to reduce the lung tumorigenesis, and to be used as a source for developing dietary supplements or nutraceuticals for reducing the risk of lung cancer among high-risk populations.

## 1. Introduction

Lung cancer is the most commonly diagnosed cancer (11.6% of all the cancers), and the leading cause of cancer deaths (18.4% of all cancer deaths) among both men and women worldwide. Among western populations, over 80% of lung cancer incidence is attributed to tobacco smoking [1]. Tobacco-specific nitrosamine, 4-(methylnitrosamino)-1-(3-pyridyl)-1-butanone (NNK), is a known lung carcinogen that causes lung tumors in laboratory animals and is likely to cause lung cancer in humans [2,3]. In the lungs, NNK is converted into reactive electrophilic metabolites, which cause point mutations in critical genes that involve cellular functions. Hence, NNK can deregulate the cell cycle, apoptosis, and DNA damage repair [4,5,6]. NNK also activates cell growth and proliferation signaling cascades, such as extracellular signal-regulated kinase 1/2 (ERK1/2), mitogen-activated protein kinase (MAPK) [5], and phosphatidylinositol 3-kinase (PI3K)/protein kinase B (AKT) [7], resulting in tumorigenesis.

Numerous *in vitro*, *in vivo,* and epidemiological studies, have reported the benefits of flavonoids and flavonoid-rich plant extracts in preventing or curing cancer, including lung cancer [8,9]. For example, oral administration of anthocyanin-rich pomegranate juice reduces lung tumorigenesis in mice by inhibiting ERK1/2 and PI3K/AKT [10]. Anthocyanin is also able to suppress MAPK, Wnt/β-catenin signaling, and induce apoptosis [11,12,13]. Haskap (*Lonicera caerulea* L.), also known as blue honeysuckle, is a berry fruit with abundant anthocyanin, particularly cyanidin-3-*O*-glucoside (C3G). Haskap berry has a higher antioxidant capacity than other common fruits [14,15]. Recent studies have demonstrated anti-inflammatory [16,17], antiarthritis [18], antiobesity [19], and antidiabetic [20] properties of haskap berry. We have demonstrated that C3G-rich haskap berry extract can reduce NNK acetate-induced DNA double-strand breaks and oxidative stress in healthy human bronchial epithelial (BEAS-2B) cells *in vitro* [21]. The objective of this study was to investigate the chemopreventive ability of lyophilized C3G-rich whole haskap berry powder (C3G-HB) against NNK-induced lung tumorigenesis in A/JCr mice. The number of tumors, tumor area, and proliferative markers were used as parameters to detect the chemopreventive ability of C3G-HB. We found that C3G-HB can suppress NNK-induced lung tumorigenesis *in vivo*.

## 2. Results

Two mice from groups C3G-HB supplemented diet continuously before and after NNK-injection (conti.-C3G-HB) and C3G-HB supplemented diet only after NNK-injection (post-C3G-HB) were euthanized due to weight loss and eliminated from the study. Observations for symptoms of stress, i.e., changes in fur color or texture, food consumption, and behavioral abnormalities such as hunched posture, fast movements, and vocalization, were performed daily.

### 2.1. The Composition of C3G-HB

The nutritional composition of the C3G-HB cv. Tundra is presented in Table 1. C3G-HB is rich with proteins (68%), fat (3.3%), and fiber (8%). The C3G content of the studied haskap berry sample was 3.4%. Additionally, C3G-HB was rich in minerals, particularly manganese, magnesium, zinc, and copper (Table 1).

### 2.2. The Response of Mice to C3G-HB Supplementation and NNK-Injection

**Figure 1:**
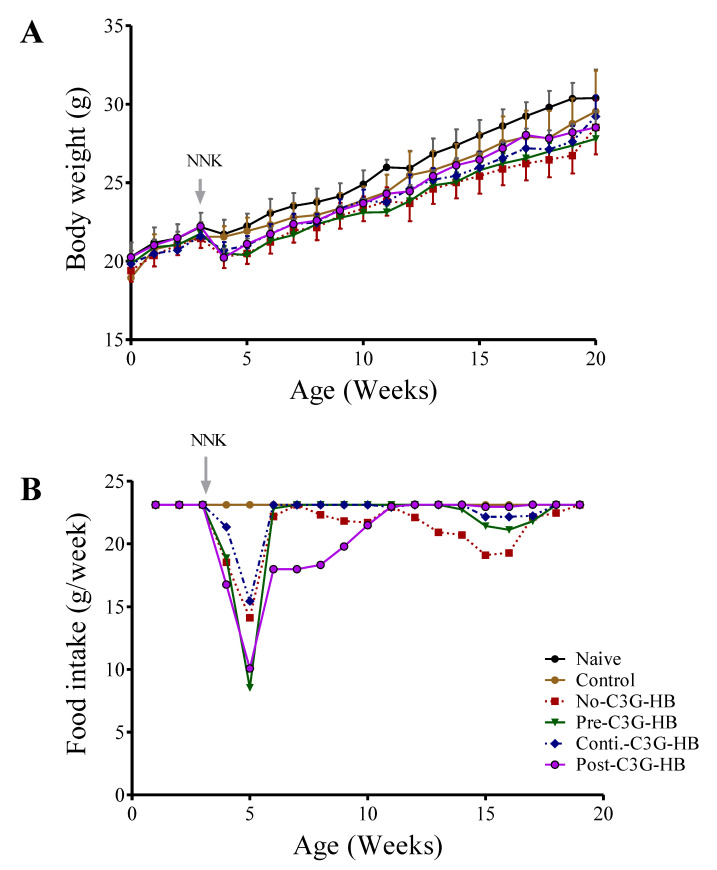
The effect of cyanidin-3-*O*-glucoside (C3G)-HB and 4-(methylnitrosamino)-1-(3-pyridyl)-1-butanone (NNK) injection on the body weight of A/JCr mice. Naive mice (*n* = 5) and NNK-injected mice in group no-C3G-HB (*n* = 10) were fed a regular mouse diet. Mice in the control (*n* = 5) and NNK-injected (pre-C3G-HB, conti.-C3G-HB, and post-C3G-HB) (*n* = 10) groups were fed with the C3G-HB supplemented diet as presented in Figure 5. (**A**) Average body weight of the mice and (**B**) Average food intake over the experimental period. The effect of C3G-HB dietary supplement and NNK carcinogen injection on the body weight of mice was determined by paired *t*-test at α = 0.05. No-C3G-HB, not given C3G-HB supplemented diet; Pre-C3G-HB, C3G-HB supplemented diet only before NNK injection; Conti.-C3G-HB, C3G-HB supplemented diet continuously before and after NNK injection; Post-C3G-HB, C3G-HB supplemented diet only after NNK injection.

Dietary supplementation of C3G-HB and NNK injection affects the body weight of mice (Figure 1A). Body weight of naive mice was significantly higher (paired *t*-test, *p* < 0.0001) in comparison to the control and no-C3G-HB groups. For instance, at the termination, the body weight of mice given C3G-HB supplement and NNK was reduced by 3.5% (naive vs. control) and 7.6%, respectively (naive vs. no-C3G-HB). Conversely, long-term C3G-HB supplementation significantly (*p* < 0.001) increased the body weight of NNK-injected mice by 2% (no-C3G-HB vs. conti.-C3G-HB), and 3.2% (no-C3G-HB vs. post-C3G-HB), respectively (Figure 1A). In fact, the weight loss in NNK-injected, no-C3G-HB mice could be linked with their dietary intake as no-C3G-HB group had significantly lower feed intake compared to the control (paired *t*-test, *p* < 0.0035) (Figure 1B).

### 2.3. Lung Tumorigenesis and Tumor Incidence

**Figure 2:**
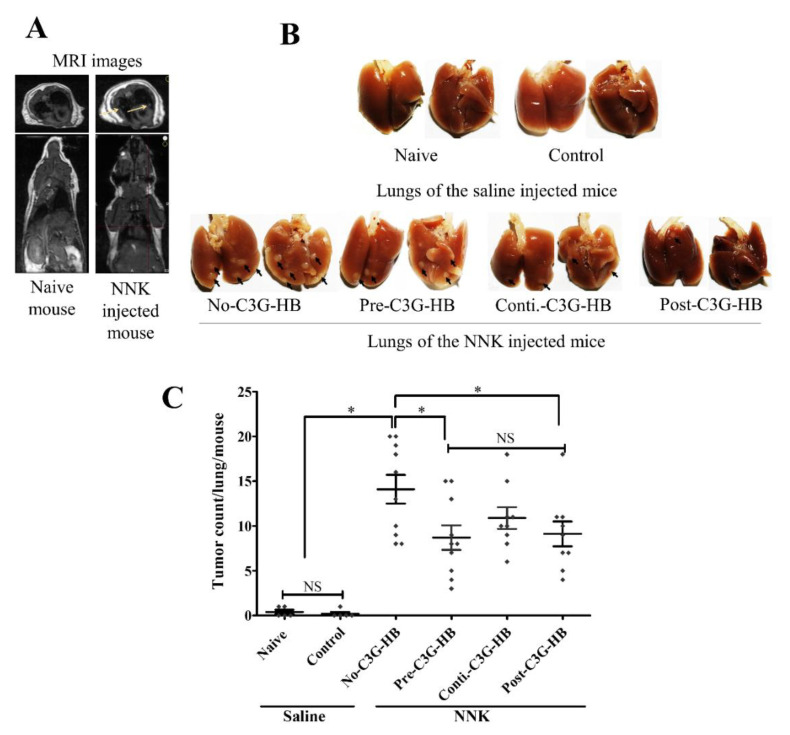
The effect of C3G-rich haskap berry supplement (C3G-HB) on NNK-induced lung tumorigenesis in A/JCr mice. Saline was injected as a sham for mice in naive and control groups. A single intraperitoneal injection of NNK (100 mg/kg body weight) was used to induce lung tumors in the rest of the mouse groups (pre-C3G-HB, conti.-C3G-HB, and post-C3G-HB). Naive mice were fed a regular mouse diet. Mice in control and NNK-injected groups were fed the C3G-HB supplemented diet, as presented in Figure 5. (**A**) The presence of lung tumors was confirmed by PET/MRI scan and a representative comparison between naive and no-C3G-HB groups (*n* = 3). (**B**) The number of peripheral tumors was counted in each lung under a dissecting microscope (*n* = 5 for naive and control groups, and *n* = 10 for NNK-injected groups). (**C**) The effect of C3G-HB dietary supplement on lung tumor multiplicity was analyzed by one-way ANOVA with Dunnett’s test at α = 0.05. No-C3G-HB, not given C3G-HB supplemented diet; Pre-C3G-HB, C3G-HB supplemented diet only before NNK injection; Conti.-C3G-HB, C3G-HB supplemented diet continuously before and after NNK injection; Post-C3G-HB, C3G-HB supplemented diet only after NNK injection. * Indicate statistical difference at *p* ≤ 0 05 with mean ± SD. NS, Results do not significantly different.

PET/MRI images confirmed the presence of tumors in the lungs of NNK-injected mice (Figure 2A). The effect of C3G-HB dietary supplementation on lung tumorigenesis was determined by the number of peripheral lung tumors (Figure 2B). The group no-C3G-HB mice, injected with NNK, and fed the control diet, had an average of 14.1 ± 1.7 tumors/mouse. NNK-injected mice in group pre-, conti.-, and post-C3G-HB, that were given the C3G-HB supplement had 8.7 ± 1.4, 10.2 ± 1.2, and 9.1 ± 1.4 tumors/mouse, respectively, and a reduction of tumor multiplicity by 38.3%, 22.8%, and 35.4%, respectively, in comparison to the no-C3G-HB group (Figure 2C). The inhibition of lung tumor multiplicity by continuous dietary supplementation of C3G-HB (conti.-C3G-HB) was not statistically significant (*p* > 0.05) from no-C3G-HB group.

The tumor incidence was not affected by the consumption of the C3G-HB dietary supplement. The tumor incidence of NNK-injected mice was 100% (10/10 and 9/9). Untreated mice (saline-injected) in naive group; 2 out of 5 mice (0.4 ± 0.2) and control; 1 out of 5 mice (0.2 ± 0.2) showed one “spontaneous” tumor on their lungs.

### 2.4. Lung Tumor Area

**Figure 3:**
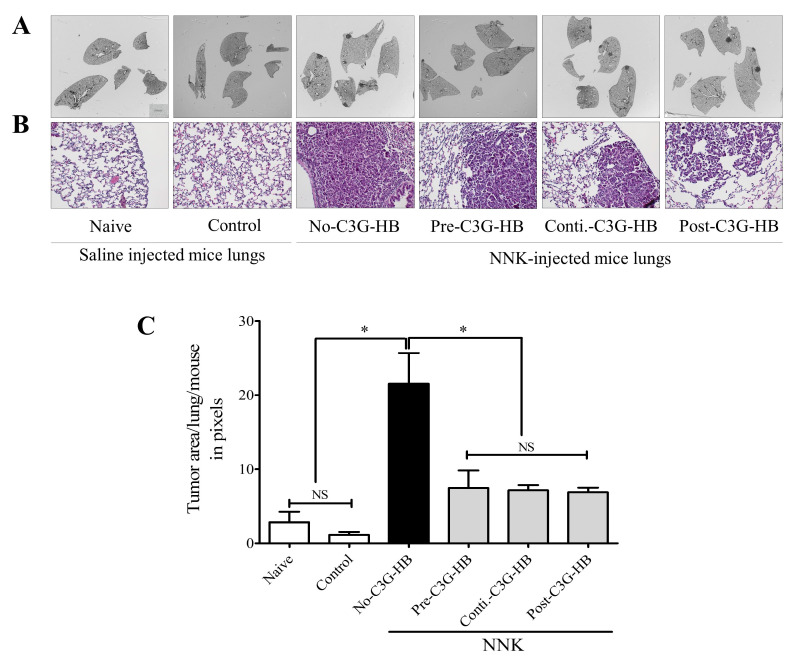
The effect of C3G-HB dietary supplementation on lung tumor area in A/JCr mice. Lung tumors were induced by a single intraperitoneal injection of NNK (100 mg/kg body weight). Saline was injected as a sham for the mice in naive and control groups. Naive mice were fed a regular mouse diet. Mice in control and NNK-injected groups were fed the C3G-HB supplemented diet, except for the mice in no-C3G-HB group (refer to Figure 5). Formalin-fixed lung sections were stained with H and E (3 sections/mouse). (**A**) The whole lung area was imaged, and the tumor area was measured by ImageJ software. (**B**) Representative H and E-stained sections, 200× magnification. (**C**) One-way ANOVA with Dunnett’s test at α = 0.05 was used for data analysis to compare the treatment effect. No-C3G-HB, not given C3G-HB supplemented diet; Pre-C3G-HB, C3G-HB supplemented diet only before NNK injection; Conti.-C3G-HB, C3G-HB supplemented diet continuously before and after NNK injection; Post-C3G-HB, C3G-HB supplemented diet only after NNK injection. * Indicate statistical difference at *p* ≤ 0 05 with mean ± SD. NS, Results do not significantly different.

The lung tumor area was measured in three consecutive lung sections, representing three depths (top, middle, and bottom) of the lungs (Figure 3). The H and E-stained sections revealed the internal tumor area and tumor characteristics. Tumor lesions were less differentiated and composed of cells with higher nuclear crowding and cytological atypia. The H and E-stained sections indicated a significantly higher (*p* < 0.0001) tumor area in the no-C3G-HB group that received NNK and the control diet. The tumor area in each section was calculated using ImageJ software. The tumor burden in NNK-injected mice was 21.6 ± 4.1. Tumor area was significantly reduced in NNK-treated mice that received the C3G-HB-supplemented diet; 7.6 ± 2.8 (pre-C3G-HB), 7.1 ± 0.6 (conti.-C3G-HB), and 6.9 ± 0.6 (post-C3G-HB), and accordingly reduced by 64.7%, 67.3%, and 68.1%, respectively.

### 2.5. Expression of PCNA and Ki-67

**Figure 4:**
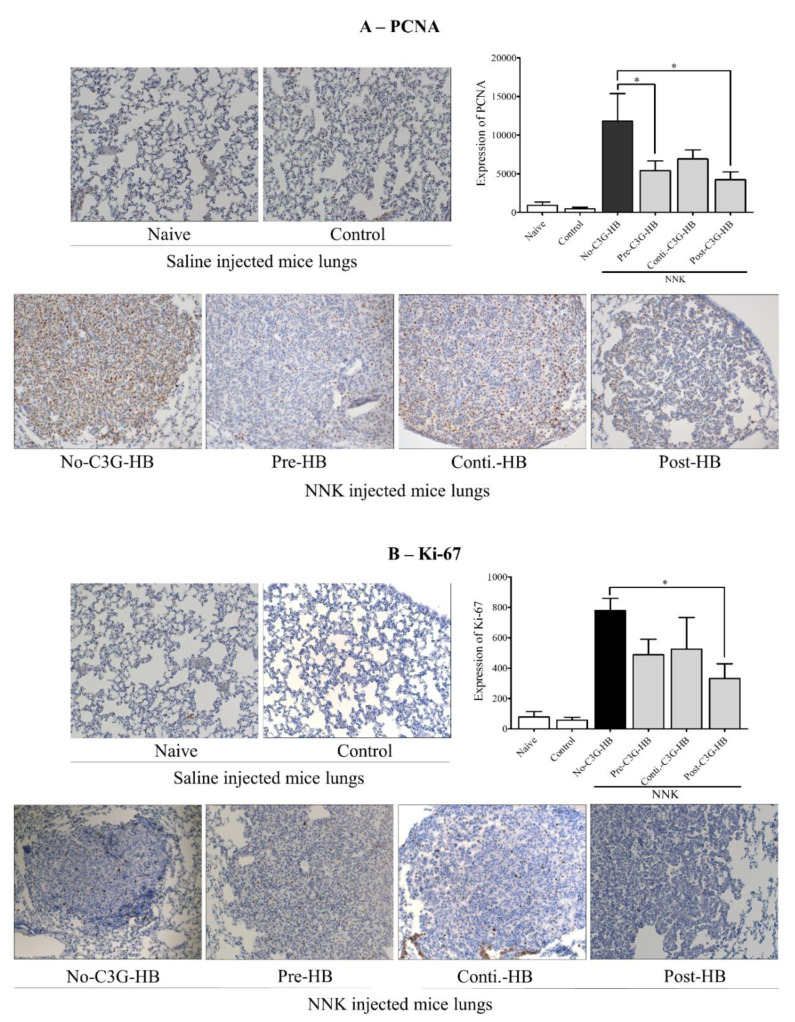
The expression of PCNA and Ki67 in lung tumors. Tumors were imaged using Zeiss Axioplan II and Axiocam HRC color camera at 200 × magnification. The number of (**A**) PCNA and (**B**) Ki-67 positive cells in each section (2 sections/mouse, *n* = 5) were counted using ImageJ software and presented as the expression in bar graphs. One-way ANOVA with Dunnett’s test at α = 0.05 and α = 0.1 was used for data analysis. No-C3G-HB, not given C3G-HB supplemented diet; Pre-C3G-HB, C3G-HB supplemented diet only before NNK injection; Conti.-C3G-HB, C3G-HB supplemented diet continuously before and after NNK injection; Post-C3G-HB, C3G-HB supplemented diet only after NNK injection. * Indicate statistical difference at *p* ≤ 0.05 with mean ± SD. NS, Results do not significantly different.

The expression of proteins involved in cell proliferation, PCNA and Ki-67, was determined in lung tissue (Figure 4). The cell proliferation markers, PCNA and Ki-67, were highly expressed (*p* < 0.0001) in the lungs of NNK-injected mice (no-C3G-HB, pre-C3G-HB, conti.-C3G-HB, and post-C3G-HB) relative to the saline-injected control mice (naive and control groups). The expression of PCNA was significantly higher compared to that of Ki-67. The level of PCNA and Ki-67 was significantly (*p* < 0.0001) reduced in the lungs of NNK-injected mice that were fed C3G-HB. As a percentage, the expression of PCNA was decreased by 41% to 64% (Figure 4A) and Ki-67 by 33% to 57% (Figure 4B), respectively. The results indicate a reduction of cell proliferation rate in lung tumors of mice fed with C3G-HB dietary supplement.

## 3. Discussion

We administered C3G-HB as a dietary supplement before, during, or after exposure to the pro-carcinogen NNK to evaluate the chemopreventive and chemotherapeutic effect of C3G-HB against NNK-induced lung tumorigenesis in A/JCr mice. The A/JCr mouse is recognized as an *in vivo* model for investigating carcinogen-induced lung tumorigenesis [22]. These mice develop spontaneous lung tumors over time [23]; hence, tumor observation in the naive (2/5 mice) and control (1/5 mice) groups is not surprising. To the best of our knowledge, this is the first study to investigate the chemopreventive effect of C3G-HB against NNK-induced lung tumorigenesis in vivo. 

C3G is the most predominant anthocyanin in haskap berry extract. C3G represents about 90% of anthocyanins in haskap berry. Our primary goal is to develop a nutraceutical from haskap berry for use in preventing lung carcinogenesis. Developing pure C3G as a nutraceutical is not practical due to the cost of purification and loss of consumer perception as a natural health product. Hence, we did not test the effect of pure C3G; however, our previous *in vitro* study confirmed that pure C3G has a similar effect as of the extracts of the C3G-HB, and reduced the carcinogen-induced DNA damage and oxidative stress in BEAS-2B normal lung epithelial cells [21].

C3G-HB (6 mg of C3G in 0.2 g of lyophilized whole haskap berry power/mouse/day) significantly (*p* < 0.05) reduced the NNK-induced lung tumor multiplicity, the most sensitive indicator of potency [24], by 38% (pre-C3G-HB) and 35% (post-C3G-HB), respectively. However, administering C3G-HB continuously before and after NNK injection was less effective (only 22%) compared to the pre- and post-supplementation. In contrast, measurement of tumor area in histological samples revealed over 65% reduction upon C3G-HB ingestion. Similarly, Khan and colleagues reported a reduction of benzo(a)pyrene- and N-nitroso-tris-chloroethylurea-induced lung tumors by 53% and 74%, respectively, in A/JCr mice that were fed with 0.2% *w*/*v* C3G-rich pomegranate juice [10]. At tumor initiation stage, NNK is converted into electrophilic metabolites (via CYP450s enzymes) that covalently bind with DNA to form bulky DNA adducts leading to lung tumorigenesis [25,26]. Our previous findings confirmed that ethanolic and aqueous extracts of C3G-HB can attenuate the NNK-induced DNA double-strand breaks, suppress oxidative stress, and induce DNA damage repair proteins in normal lung epithelial BEAS-2B cells [21]. Oral administration of haskap berry attenuates oxidative stress in mice [19] and restores oxidative defense mechanisms by activating catalase, superoxide dismutase, glutathione peroxidase, and glutathione in mice that were exposed to ionizing radiation [27,28,29]. In addition, flavonoids have been reported to reversibly and irreversibly inhibit cytochrome 450 (CYP450) enzymes and interfere in xenobiotic metabolism [30]. The antioxidant activity of C3G-HB [21] may have attenuated the NNK-related electrophilic metabolites and hence inhibited the formation of DNA adducts that trigger lung tumorigenesis. Therefore, it is crucial to investigate the oxidative defense mechanism of the C3G-HB and its effect on CYP450 enzyme activity in the future. 

PCNA is necessary for DNA synthesis (a processivity factor of DNA polymerases) and DNA repair (involved in nucleotide excision repair and base excision repair). PCNA is highly expressed during the active cell cycle; G_1_ phase, peaks at S-phase and declines during G_2_/M-phases. Ki-67 is expressed in G_1_-, S-, and G_2_-phases, but not in the G_0_-phase of the cell cycle [31]. Similar to previous findings [3,4,5], NNK induced lung cell proliferation, which is indicated by the highly expressed PCNA (12.7-fold) and Ki-67 (10-fold) in lung tissues of the no-C3G-HB group comparison to the naive group. The C3G-HB dietary supplementation reduced cell proliferation markers, PCNA (40–60%) and Ki-67 (30–60%), respectively. C3G, as a pure compound (250 and 500 µM) and in fruit extracts (0.2% *w*/*v* C3G-rich pomegranate juice) inhibits cell proliferation through deactivating MAPK and PI3K/AKT signaling pathways, which are activated by NNK at cancer progression [10,13,32]. Thus, it is necessary to study the effect of C3G-HB in cell proliferation pathways to understand its tumor inhibitory mechanism. 

Regardless of the feed intake, the C3G-HB supplement reduced the body weight of the mice by 3.5% comparison to the regular diet (control vs. naive). C3G enhances energy metabolism by upregulating brown adipose tissue mitochondrial function [33]. Ingestion of C3G-rich blood orange juice [34] and purple sweet potato [35] results in reduced lipogenesis, including triglycerides through activation of adenosine monophosphate-activated protein kinase (AMPK) signaling pathways. Therefore, enhanced metabolism and/or reduction of fat synthesis might account for the weight loss effect of C3G-HB in control mice over the naive group. AMPK is also identified as a metabolic tumor suppressor which reprograms cellular metabolism and prevent tumorigenesis [36]. Therefore, C3G-activated AMPK may have regulated energy levels that inhibit cell proliferation. These results suggest the beneficial health effect of C3G-HB against NNK-induced lung tumorigenesis *in vivo*.

In humans, C3G metabolism generates various metabolites such as C3G glucuronides, methylates of the C3G, i.e., peonidin-3-glucoside, and simple phenolic acids including protocatechuic acid, ploroglucinaldehyde, and hippuric acid [37,38,39]. Even though the metabolites of C3G are similar in humans and mice, the clearance rate of C3G in humans is slower than in mice. In humans, C3G metabolites are present in the blood plasma for ≤ 48 h. In contrast, in mice, a major fraction of C3G metabolites are excreted after 24 h [40,41]. Therefore, when considering C3G-HB as a nutraceutical for use in humans, the absorption, distribution, metabolism, and elimination of C3G should be evaluated using a properly designed human clinical study. 

## 4. Materials and Methods

### 4.1. Materials 

This study was performed at Dalhousie University’s animal care facility, following the approval of the University Committee on Laboratory Animals (protocol 15–106). The carcinogen, 4-(methylnitrosamino)-1-(3-pyridyl)-1-butanone (NNK, MW. 207.23 g/mol, Cat No. M325750) was purchased from Toronto Research Chemicals Inc., Toronto, ON, Canada. Frozen haskap berry cv. Tundra was obtained from LaHave Natural Farm, Blockhouse, NS, Canada. Female A/JCr albino mice at 3–4 weeks age (n = 50) were purchased from Charles River Laboratories, Inc., Montreal, QC, Canada.

### 4.2. Preparation of C3G-HB and Analysis

Frozen haskap berries were lyophilized, ground to a fine powder, and stored at −80 °C. A representative sample was analyzed to determine the nutrient composition (Harlow Institute, Department of Agriculture, Truro, NS, Canada). C3G was quantified by high-performance liquid chromatography and mass spectrophotometry (HPLC/MS/MS, Waters Limited, Mississauga, ON, Canada) after extraction (1 mg/mL) using methanol containing 1% acetic acid and filtered through a 0.22 µm nylon filter [20]. 

### 4.3. Preparation of Dietary Supplement

Ingestion of 1.5 g polyphenols/day for a healthy adult of 70 kg body weight is considered to be a health-promoting dose [42,43]. A health-promoting C3G-HB dose, equivalent to the animal dose, was calculated as follows [44]:

$$\mathrm{Human}\;\mathrm{equivalent}\;\mathrm{dose}\;(\mathrm{mg}/\mathrm{kg})=\mathrm{Animal}\;\mathrm{dose}\;(\mathrm{mg}/\mathrm{kg})\times \frac{\mathrm{Animal}\;\mathrm{Km}\;\mathrm{Factor}*}{\mathrm{Human}\;\mathrm{Km}\;\mathrm{Factor}}$$

Km factor = body weight (kg)/body surface area (m^2^). The Km factors of mouse and adult human are 3 and 37, respectively [44].

Accordingly, the experimental diet/mouse/day consisted of 0.2 g C3G-HB (equivalent to 6 mg of C3G/mouse/day) and 5% Splenda® mixed into regular mouse chow (Prolab® RMH 3000 from LabDiet, St. Louis, MO, USA) and formed into a 2 g (dry weight) pellet. The control diet consisted of regular mouse chow containing 5% Splenda^®^. C3G-HB powder was mixed thoroughly for 20 min to obtain a homogeneous preparation for use in making pellets. Pellets were prepared every two days and stored in sealed containers in the dark at 4 °C.

### 4.4. Experimental Plan and Procedure 

**Figure 5:**
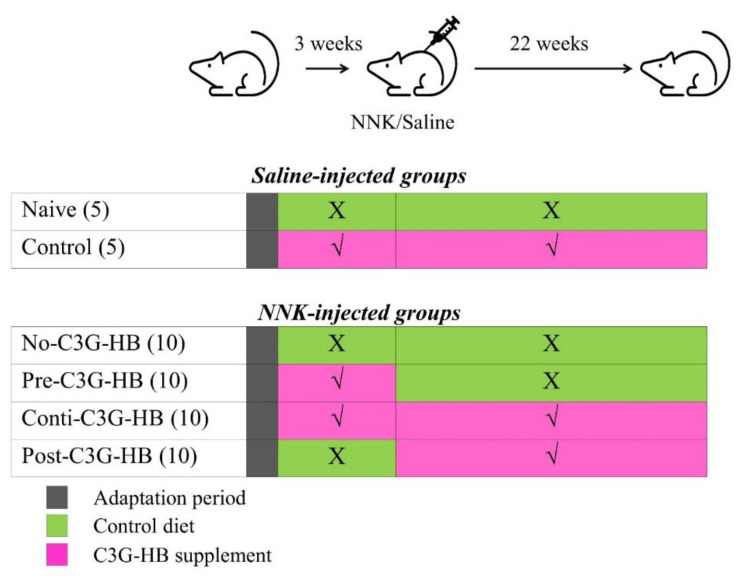
Experimental protocol for investigating the chemopreventive ability of C3G-HB in NNK-induced lung tumorigenesis in A/JCr mice. NNK (100 mg/kg body weight/mouse) or saline was injected three weeks after the adaptation period; *n* = 5 in saline-injected groups, naive and control, and *n* = 10 in NNK-injected groups (no-C3G-HB, pre-C3G-HB, conti.-C3G-HB and post-C3G-HB). No-C3G-HB, not given C3G-HB supplemented diet; Pre-C3G-HB, C3G-HB supplemented diet only before NNK injection; Conti.-C3G-HB, C3G-HB supplemented diet continuously before and after NNK injection; Post-C3G-HB, C3G-HB supplemented diet only after NNK injection.

The experiment was designed to evaluate the early and late intervention of C3G-HB against NNK-induced lung tumorigenesis (Figure 5). Mice were housed individually in filter-topped plastic cages and maintained under 12-h light-dark cycles. After one week of adaptation, mice were randomly divided into six groups, n = 5 for saline-injected naive and control groups and n = 10 for NNK injected no-C3G-HB, pre-C3G-HB, conti.-C3G-HB and post-C3G-HB groups). Prolab^®^ RMH 3000 diet and distilled water were provided ad libitum. The C3G-HB or control pellets were given daily as a dietary supplement. Briefly, mice in naive and no-C3G-HB groups were given control pellets, while the diet of control, pre-C3G-HB, conti.-C3G-HB and post-C3G-HB groups was supplemented with C3G-HB (Figure 5). Mice in pre-C3G-HB were fed the C3G-HB supplemented diet for three weeks, prior to NNK injection and then switching to the control diet after NNK injection. Post-C3G-HB group was given the control diet until the NNK injection and then switched to C3G-HB supplemented diet until the end of the experiment. Three weeks after the start of dietary supplementation, a single dose of NNK (100 mg/kg body weight in 0.2 mL saline) was injected into the peritoneal cavity of mice to induce lung tumors. Mice in naive and control groups, received an equivalent volume of saline. Once a week, body weight was measured until the end of the experiment. 

### 4.5. Lung Tumor Assays

#### 4.5.1. Positron Emission Tomography-Magnetic Resonance Imaging (PET-MRI)

Before euthanizing mice, lung tumorigenesis was confirmed by PET-MRI at the Biomedical Translational Imaging Center (BIOTIC) at the IWK Children’s Hospital, Halifax, NS, Canada. Briefly, three mice from naive and no-C3G-HB groups were randomly selected and fasted for six hours and then injected with ^18^F-fluorodeoxyglucose 100 μϹi via the tail vein. After 30 min, the mice were anesthetized, and a PET-MRI scan was performed. Breathing pattern, heart rate, and body temperature were monitored throughout the scanning period. 

Twenty-two weeks after NNK treatment, mice were anesthetized with isoflurane. Blood samples were collected by cardiac puncture, and a higher dose of isoflurane was used to sacrifice mice. Dissected lungs were perfused and washed in phosphate-buffered saline (PBS) before being fixed in 10% (*v*/*v*) acetate-buffered formalin. Peripheral lung tumors were enumerated using a dissecting microscope. Lungs were embedded in paraffin, and paraffin-embedded tissues were stored for histopathological examination. 

#### 4.5.2. Tumor Histology and Tumor Area

Paraffin-embedded lungs were cut into 5 µm thick sections (50 tissue sections/lung) using a microtome (Leica Rm 2255, Leica Biosystems, Concord, ON, Canada). Three sections representing three areas of the lungs were selected at predetermined depths and stained with hematoxylin and eosin (H and E). The H and E-stained lung sections were imaged under bright field microscopy (AxioPlan 11MOT AxioCam HRc, Carl Zeiss Canada Ltd., Toronto, ON, Canada), and lung tumor area was quantified using ImageJ software [45]. 

#### 4.5.3. Immunohistochemistry 

The expression of proliferating cell nuclear antigen (PCNA) and Ki-67 was evaluated by immunohistochemistry (IHC). Briefly, paraffin sections were deparaffinized in xylene, rehydrated through an ethanol solution gradient and washed carefully in running tap water. Antigen retrieval was carried out by heating sections in 0.01 M citrate buffer (pH 6.0) for 30 min in the decloaking chamber. Endogenous peroxidase activity was quenched by incubating the section in 3% H_2_O_2_ in Tris-buffered saline (TBS) for 10 min. Non-specific binding sites were blocked by incubation with rodent block (M) from Biocare Medical, Pacheco, CA, USA. The sections were incubated overnight at room temperature in a humid chamber with monoclonal antibodies against PCNA (1:6000 dilution) or Ki-67 (1:50 dilution). After several washes with TBS, the slides were incubated with anti-rabbit horseradish peroxidase-conjugated secondary antibody (EnVision, Dako North America Inc.Carpinteria, CA, USA) for 30 min, then washed three times with TBS and incubated with chromogen 3-diaminobenzidine (DAB Chromogen kit, Biocare Medical, Pacheo, Ca, USA) for 3 min. The slides were carefully rinsed under running tap water and counterstained with hematoxylin. The slides were observed under bright field microscopy (AxioPlan 11MOT AxioCam HRc, Carl Zeiss Canada Ltd., North York, ON, Canada) at 200× magnification.

### 4.6. Statistical Analysis

The observed differences in the tumor multiplicity, tumor area, PCNA and Ki-67 expression were tested for statistical significance using one-way Analysis of Variance (ANOVA). Tukey’s pairwise comparison and Dunnett’s test with a 95% confidence interval was used for comparisons among multiple groups. Minitab statistical software was used for data analysis.

## 5. Conclusions

In summary, we have demonstrated that dietary supplementation of C3G-HB can inhibit the NNK-induced lung tumorigenesis in A/JCr mice. C3G-HB may be a promising dietary supplement to suppress lung cancer development among high-risk populations such as smokers, possibly via effects on critical cellular signaling pathways that regulate cell proliferation. Future studies of the effects of C3G-HB on phase I and phase II metabolic enzymes and cell signaling pathways will elucidate the mode of action of C3G-HB against lung carcinogenesis.

## Figures and Tables

**Table 1:** The nutritional composition of lyophilized powder of haskap berry cv. Tundra.

Nutrient	%	Mineral Content	%
Dry matter	91.64	Potassium	1.175
Protein digestibility	67.76	Magnesium	0.056
Crude protein	4.86	Phosphorous	0.176
Bound protein	5.10	Calcium	0.105
ADIN	0.25	Sodium	0.016
Crude fat	3.33	Copper (mg/kg)	6.34
Acid detergent fiber	3.64	Manganese (mg/kg)	<10.00
Neutral detergent fiber	4.28	Zinc (mg/kg)	7.74
Ash	2.57	Cyanidin-3-*O*-glucoside	3.4

All the parameters are presented in percentages unless the unit is indicated in front of the parameter in the table. ADIN, acid detergent insoluble nitrogen.
